# Synergistic Effect of Iontophoresis and Motor Imagery Training on Upper Limb Functional Reach in Subacute Stroke Patients

**DOI:** 10.7759/cureus.109121

**Published:** 2026-05-18

**Authors:** Yukti K Agrawal, Suraj Kanase

**Affiliations:** 1 Department of Neurosciences, Krishna College of Physiotherapy, Krishna Vishwa Vidyapeeth (Deemed to be University), Karad, IND

**Keywords:** arm function, functional recovery, motor relearning, muscle tone, upper limb rehabilitation

## Abstract

Introduction

Stroke is a leading cause of long-term disability, with upper limb dysfunction being one of the most common impairments affecting independence in daily activities. Reduced functional reach is often associated with spasticity, muscle weakness, and impaired motor planning. Conventional physiotherapy primarily focuses on physical rehabilitation but may not sufficiently address both peripheral and central mechanisms involved in recovery. Therefore, combining peripheral interventions such as iontophoresis with central strategies like motor imagery (MI) may enhance functional outcomes. This study aimed to evaluate the synergistic effect of iontophoresis combined with MI training on upper limb functional reach in subacute stroke patients.

Methods

This experimental study included 34 subacute stroke patients (40-60 years) randomly allocated into Group A (conventional therapy with magnesium sulphate iontophoresis) and Group B (conventional therapy with iontophoresis plus MI). Interventions were administered for six weeks (five sessions/week). Outcome measures included the Functional Reach Test (FRT), Modified Ashworth Scale (MAS), and Fugl‑Meyer Assessment (FMA)-upper‑limb component. Pre‑ and post‑intervention data were analyzed using paired and unpaired t‑tests.

Results

Both groups showed significant post‑intervention improvements; however, Group B demonstrated significantly greater gains. The mean FRT improved by 11.18 cm in Group B compared to 2.31 cm in Group A (p < 0.0001). MAS and FMA scores also showed highly significant intergroup differences (p < 0.0001), indicating greater reduction in spasticity and improvement in voluntary motor control with the combined approach.

Conclusion

The addition of MI to iontophoresis and conventional therapy produces superior improvement in upper‑limb functional reach and motor recovery in subacute stroke patients, offering a feasible and cost‑effective neurorehabilitation strategy.

## Introduction

Hemiplegia involves impairment of one side of the body, which can present with the arm affected more than the leg, or with the leg affected as much as or more than the arm [1]. It is among the most common impairments following a stroke and significantly contributes to walking difficulties. A study conducted in 2023 in India reported an incidence rate of stroke ranging from 116 to 163 per 100,000 people. Approximately 66% of the Indian population resides in rural areas, and among these rural inhabitants, about 71% of stroke-related deaths have occurred, highlighting a disproportionately high number of fatalities [2].

The prevalence rates of stroke in rural India were found in studies that included individuals of all ages, ranging from 55 to 388.4 per 100,000 people. The most common type of impairment was motor-related, with over 80% of patients experiencing some form of motor impairment. This included facial palsy (50%), dysarthria (47%), limb weakness (30%), and ataxia (17%), with similar frequencies observed for weakness in both upper and lower limbs, as well as between the right and left sides [3].

Upper-limb dysfunction affects a large proportion of stroke survivors. Estimates from contemporary reviews place motor dysfunction in roughly half to three-quarters of patients after stroke, with upper-limb involvement reported in up to 70-85% during the acute phase and persisting in many into the chronic stage. Impairments relevant to functional reaching include shoulder-elbow weakness, loss of inter-joint coordination, delayed motor activation, spasticity that constrains the movement path, and somatosensory deficits that impair endpoint control and error correction. These deficits not only reduce reach distance but also increase compensatory trunk movements and reduce movement smoothness and functional task success [4]. Spasticity in the biceps brachii muscle is one of the reasons for the limitation of functional reach of the upper limb. Following injury to the central nervous system, muscle tone is abnormal, characterized by a loss of inhibition and excess excitation of muscles. This creates an uneven balance between both types of input from the central nervous system and therefore causes hypertonicity. Hypertonicity may also be caused by a physical or chemical irritant acting on the muscle's nerve fibers, which ultimately causes the same excess muscle tightness as described above [5].

A wide range of interventions has been trialed to improve upper-limb reach after stroke. Conventional physiotherapy and occupational therapy remain foundational, emphasizing task-specific practice, strengthening, and range-of-motion exercises. Over the past two decades, more targeted or high-intensity strategies, constraint-induced movement therapy (CIMT), robotic assistance, functional electrical stimulation (FES), task-oriented training, virtual reality, and mirror therapy, have shown benefits in selected populations [6,7]. Yet despite promising results, several limitations persist: many approaches require specialized equipment or intensive therapist time (restricting scalability), gains on impairment-level measures do not always translate to improved real-world arm use, heterogeneity of protocols produces inconsistent effect sizes across studies, and pain or spasticity can limit participation. Moreover, some techniques (e.g., high-intensity or device-based interventions) are less accessible in low-resource settings. These gaps create an opportunity for complementary, low-risk adjuncts that can enhance motor recovery mechanisms and be integrated into routine rehabilitation.

Two adjunct modalities with distinct but potentially complementary mechanisms are iontophoresis and motor imagery (MI). Iontophoresis is a noninvasive technology that uses controlled low-level electrical energy to transport ionized drugs through the skin in a safe and effective manner [8]. Magnesium sulphate has been known to be a muscle relaxant and vasodilator for over a century, and it is considered physiologically to be an antagonist of calcium. In a systematic review reported by Nepal et al., it was found that magnesium sulphate was able to relieve muscle spasms in cases of tetanus [9]. Gutiérrez-Román et al. also observed that it was relevant in anesthesiology as an adjuvant for sedation, analgesia, neuromuscular relaxation, and motor relaxation [10]. Antenatal magnesium sulfate was also associated with a significant reduction in the risk of cerebral palsy by preventing preterm birth [11]. There are also reports of effectiveness in treating myalgias, neuritis, deltoid bursitis, and low back pain spasm [12].

MI is a cognitive process that involves mentally simulating a movement without any physical execution. It represents the internal rehearsal of a motor act under conscious control, activating neural networks similar to those engaged during actual movement [13]. MI enhances motor planning, proprioceptive prediction, and motor learning, supporting rehabilitation when combined with physiotherapy. Studies show that structured MI training improves movement imagery ability, muscle activation patterns, and functional mobility in neurological populations such as cerebral palsy and stroke [14,15]. Clinical trials and meta-analyses indicate that MI, when combined with conventional therapy, can improve motor outcomes after stroke, particularly for upper-limb function, and it is inexpensive, safe, and easy to scale (including for home practice). MI’s principal advantage is its ability to drive central plasticity even when physical practice is limited by weakness or fatigue [16]. 

A synergistic rationale supports combining iontophoresis and MI for subacute stroke rehabilitation of reach. By reducing peripheral pain and muscle spasticity with iontophoretic delivery of targeted agents, patients may achieve greater tolerance for active reaching practice; simultaneous or sequential MI can prime motor networks and reinforce the cortical representations necessary for improved reach kinematics. Together, these interventions target both peripheral and central constraints on reach, a biopsychosocial approach that could enhance transfer from impairment-level change to functional arm use. Given the pressing need for scalable, evidence-based strategies that are feasible in diverse clinical settings, exploring whether a combined iontophoresis and MI protocol produces additive or synergistic improvements in functional reach is both clinically important and scientifically timely.

## Materials and methods

This pre-post experimental study was conducted over a period of six months from 10/10/2025 to 10/04/2026 at the Neurosciences Outpatient Department, Krishna College of Physiotherapy, Karad, Maharashtra, after obtaining institutional ethical clearance. This study was prospectively registered with the Clinical Trials Registry of India (CTRI) under registration number CTRI/2026/04/109733. Thirty‑four subacute stroke patients diagnosed by a neurologist were recruited using a convenience sampling method. Participants aged 40-60 years with stroke duration between two weeks and six months, mild to moderate elbow flexor spasticity (Modified Ashworth Scale (MAS): 1 to 2), voluntary upper‑limb movement, stable blood pressure, and adequate cognition (Mini-Mental State Examination: ≥24) [17] were included. Patients receiving antispasticity medications, those with severe spasticity or flaccidity, co‑morbid medical conditions, metallic implants, pacemakers, or allergy to magnesium sulphate were excluded.

Participants were randomly allocated into two groups using a simple random allocation using sealed opaque envelopes. Interventions were administered five times per week for six weeks.

Group A (conventional therapy)

Participants received conventional upper limb rehabilitation along with iontophoresis using 5% magnesium sulphate over the biceps brachii muscle. The current density was maintained between 0.1 and 0.2 mA/cm², gradually increasing the duration from 10 minutes in the first week to 20 minutes by the fourth week. Treatment was given five times per week for six weeks, accompanied by conventional exercises such as active-assisted movements, bilateral reaching, fine motor activities, and task-oriented functional training.

Group B (experimental group)

Participants in this group received the same conventional therapy and iontophoresis protocol as Group A, along with an additional MI training component. The duration of MI progressively increased from 15 minutes in the first week to 30 minutes by the fourth week, combined with visualization of functional reaching and daily living activities. Sessions were conducted five times per week for six weeks, integrating both physical and mental practice to enhance upper limb functional reach.

Outcome measures (Functional Reach Test (FRT), MAS, Fugl-Meyer Assessment (FMA) scale (arm component) were recorded pre‑ and post‑intervention. The FRT was used to assess upper-limb reaching ability and dynamic stability relevant to daily activities [18]. The MAS measured biceps spasticity, a key factor limiting reach in stroke [19]. The Fugl-Meyer Assessment (upper-limb component) evaluated motor recovery, coordination, and voluntary control [20]. Together, these validated measures captured functional performance, muscle tone, and impairment-level motor changes following the intervention. Statistical analysis was performed using paired and unpaired t‑tests, with significance set at p < 0.05.

The data were entered into an MS Excel (Microsoft Corporation, Redmond, Washington, United States) spreadsheet, tabulated, and subjected to statistical analysis. Various statistical measures, such as mean, standard deviation (SD), and test of significance, were utilized to analyze the data. The results were concluded to be statistically significant if p < 0.05. Data were analyzed using Graphpad INSTAT, trial version 3.0632 (GraphPad Software, San Diego, California, USA). A paired t-test was used to analyze the difference between pre- and postmeasurements within the group, and an unpaired test was performed to analyze the synergistic effect of iontophoresis and MI training on the functional reach of the upper limb in subacute stroke patients.

## Results

A total of 34 men and women of the age group 40-60 years having impaired functional reach of the upper limb volunteered to participate in the study, out of which five women (14.7%) and 29 men (85.3%) have completed six weeks of the program (Table 1). The age-wise distribution of subacute stroke patients showed that 11 participants were in the 46-50 years age group. Nine patients each belonged to the 40-45 and 51-55 years groups, while five patients were aged 56-60 years, indicating a predominance of middle-aged individuals in the study sample (Figure 1). 

**Table 1:** Demographic data

Variable	Frequency (n)	Percentage (%)
Gender		
Male	29	85.3%
Female	5	14.7%
Age group (years)		
40-45	9	26.5%
46-50	11	32.4%
51-55	9	26.5%
56-60	5	14.7%

Intragroup comparisons of outcome measures are presented in Table 2, showing that both Group A and Group B showed significant improvements after intervention. However, Group B demonstrated greater gains in functional reach (10.19 cm vs 2.31 cm), larger reduction in spasticity (−1.12 vs −0.56), and greater improvement in motor recovery (14.93 vs 4.37), all with stronger statistical significance.

**Table 2:** Comparison of pre- and post-intervention scores within groups (Group A & Group B) * p < 0.05 (statistically significant); ** p < 0.01 (very significant); *** p < 0.001 (highly significant); FRT: Functional Reach Test; MAS: Modified Ashworth Scale; FMA: Fugl‑Meyer Assessment Values are presented as mean ± standard deviation (SD). Within-group comparisons were analyzed using the paired t-test. A p-value of <0.05 was considered statistically significant

Outcome measure	Group	Phase	Mean	SD	t-value	p-value	Mean difference
FRT	Group A	Pre	20.68	2.02	3.12	0.003*	2.31
		Post	23	2.16			
	Group B	Pre	21.68	2.02	16.29	<0.0001***	10.19
		Post	31.87	1.85			
MAS	Group A	Pre	1.75	0.77	2.08	0.045*	-0.56
		Post	1.18	0.75			
	Group B	Pre	2.12	0.71	4.7	<0.0001***	-1.12
		Post	1	0.63			
FMA	Group A	Pre	35.81	5.55	2.29	0.029*	4.37
		Post	40.18	5.23			
	Group B	Pre	35.68	5.61	7.6	<0.0001***	14.93
		Post	50.62	5.48			

Intergroup comparisons of outcome measures are summarized in Table 3, which revealed that Group B showed significantly greater improvements than Group A across all outcomes. Functional reach improved more in Group B (10.19 cm vs 2.31 cm; p < 0.0001), spasticity reduction was greater (1.12 vs 0.56; p = 0.008), and motor recovery gains were higher (14.93 vs 4.37; p < 0.0001). These findings indicate a significantly superior effect of the combined intervention in Group B.

**Table 3:** Intergroup Analysis of outcome measures between Group A and Group B * p < 0.05 (statistically significant); ** p < 0.01 (very significant); *** p < 0.001 (highly significant); FRT: Functional Reach Test; MAS: Modified Ashworth Scale; FMA: Fugl‑Meyer Assessment Values are expressed as mean ± standard deviation (SD). Between-group comparisons were performed using the unpaired t-test on the mean change (post–pre differences). A p-value of <0.05 was considered statistically significant

Outcome measure	Group	N	Mean	SD	Mean difference	t-value	p-value
FRT	Group A	17	2.31	0.79	8.87	16.68	<0.0001
	Group B	17	10.19	1.97			
MAS	Group A	17	0.56	0.51	0.56	2.8	0.008
	Group B	17	1.12	0.61			
FMA	Group A	17	4.37	2.39	10.56	11.97	<0.0001
	Group B	17	14.93	2.59			

## Discussion

The main purpose of this study was to assess the additive benefit of combined iontophoresis and MI training on the functional reach of the upper extremity in patients with subacute stroke. The results of this study demonstrate that combined iontophoresis and MI induced a greater improvement in upper-extremity functional reach than either therapy or conventional therapy alone. While these effects are likely linked to the two interventions, they may work synergistically by providing complementary effects: iontophoresis improves peripheral impairments (spasticity and pain), while MI facilitates central motor activation (neuroplasticity).

One of the main aftereffects of stroke is hemiplegia, which is defined as having half of your body that cannot move (or you lack muscle control) due to damage caused by the stroke. This impacts your ability to move your weak side and creates an inability to move in a coordinated manner [1]. This condition contributes to long-term disability and decreased quality of life. In India, the burden of stroke is surging rapidly, with incidence estimates ranging between 116 and 163 per 100,000 people [2]. An inordinate share of stroke deaths occurs in rural communities, where access to rehabilitation services is limited [3]. Research has also shown that motor impairment, specifically weakness of the upper and lower limbs, is the most frequent residual deficit among stroke survivors [4]. Therefore, the development of accessible, affordable, and evidence-based rehabilitation approaches could be very important.

Around 70% to 85% of stroke patients still have upper-limb dysfunction during the acute stage and continue to have problems chronically [5]. Functional reaching is affected by spasticity, weakness, loss of coordination, and somatosensory feedback. Spasticity in the biceps brachii may limit elbow extension and shoulder movement, which limits reach and can contribute to compensatory trunk displacement [5]. Functional independence outcomes continue to be observed in conventional therapies such as task-oriented training, CIMT, robotic assistance, or mirror therapy, considering potential benefits. However, the application of conventional stroke therapies can require an abundance of therapist time or resources, and they may therefore not be practical in low-resource settings [6,7]. Alternative therapies such as iontophoresis and MI provide a meaningful alternative to conventional therapies.

Iontophoresis is a type of noninvasive approach to transport ionized medications through the skin using a very mild electrical current in a controlled manner. Magnesium sulfate is the substance used in this investigation, which acts as a calcium antagonist and has demonstrated its muscle relaxant and vasodilatory properties for over 100 years [9]. Studies have demonstrated its effectiveness in muscle spasms in tetanus, as an adjunct to sedation, analgesia, and neuromuscular blockade in anesthesia [10]. It has also demonstrated a decreased risk of cerebral palsy if used antenatally, and provides benefit with muscular pain, neuritis, and muscle spasm [11,12]. The present data corroborate the findings of Onigbinde et al., who found that magnesium sulfate iontophoresis substantially decreases elbow flexor spasticity poststroke [5].

The likely underlying reason for these developments is the inhibitory effect of magnesium on calcium entry at neuromuscular junctions, reducing muscle tone and excitability. This results in less resistance to stretching, and a release of hypertonia allows greater ease in movement. Iontophoresis may have supported even smoother, longer reach movements by reducing hypertonia in the upper limb. Furthermore, improved blood flow as a result of vasodilation may have contributed to reduced fatigue and increased muscle performance.

MI, on the other hand, refers to a mental practice method of imagining the process of movement without implementing the actual movement [21]. The same neural circuits that are activated in a motor execution, such as those in the premotor cortex, supplementary motor area, and cerebellum, are also activated during MI; MI is a process through which an individual simulates or imagines performing a motor task without any actual movement occurring [13]. The implication of practice in MI is to activate these brain areas and strengthen motor representation and motor planning through repeated practice, supporting neural reorganization and learning [22]. Clinical trials and meta-analysis research show that MI practice following conventional therapy improves upper-limb function, movement accuracy, and performance of daily tasks following stroke [16].

The findings in this study support those of previous studies and demonstrate that MI training improved reaching ability in stroke participants. The most likely explanation is that MI is an effective means to enhance anticipatory motor control and proprioceptive awareness. Even if physical movement is not possible, MI uses motor neurons and induces a degree of cortical plasticity, which can assist recovery.

Thus, using iontophoresis and MI together is likely to have a synergistic effect. Iontophoresis can help reduce peripheral spasticity and prepare the muscles for action (i.e., thixotropic effect), and MI activates the central motor circuits that are responsible for planning and execution of movement. Together, this provides for a comprehensive peripheral-central interaction for restoring function in upper-limb reach. When spasticity and pain are reduced, patients are likely to have more focus on imagery training and active voluntary movement practice in a way that can enhance cortical reorganization.

This integrative approach follows suggestions currently emphasized in neurorehabilitation principles that endorse multisystem engagement, using sensory, motor, and cognitive systems together, which leads to the greatest recovery. Unlike robotic or device-based interventions, which are more costly and difficult to implement technically, iontophoresis and MI are both simple, safe, and have been established to be usable in rural or lower-resource clinical environments [2,3]. Therefore, utilizing them in combination may handle both the physiological and logistical challenges in stroke rehabilitation.

The improvement in functional reach in this case series indicates the overall recovery benefit of addressing both central and peripheral interventions. Iontophoresis likely facilitated the improvement of muscle tone, and decreasing muscle tone in the labrum likely caused local decreases in resistance, and MI likely increased neural efficiency and coordination/reduced control of movement. Thus, together they may have led to improved smoothness, range, and accuracy of functional reach.

There are specific limitations acknowledged in this examination. The study was completed in a relatively short (subacute) phase of stroke recovery, which can affect the generalizability of the results. Additionally, neurophysiological assessments, such as electromyography (EMG) and functional magnetic resonance imaging (fMRI), were not included, which could have provided objective evidence of cortical reorganization in response to MI. Future studies should consider longer follow-ups and larger sample sizes to look into the long-term retention of functional gains.

Notwithstanding these shortcomings, the data highlight a feasible, low-risk intervention strategy for stroke rehabilitation. The additive effect of iontophoresis and MI addressed both cortical activation and muscle tone and was associated with improved functional reach range in patients recovering from a subacute stroke. It is also inexpensive, broadly applicable, aligned with existing physiotherapy and rehabilitation protocols, and presents a potential opportunity for hospitals or clinical environments to successfully implement within multidimensional treatment.

## Conclusions

The study concludes that MI combined with iontophoresis produced better outcomes than either treatment alone (e.g., improved the reach of their upper limb, reduced spasticity, helped recover motor function, and controlled the troublesome peripheral muscle of the arm) in stroke patients at the subacute phase. This approach is an opportunity to address limitations related to both peripheral muscle and central motor control as a result of their respective effects, which result in improved function compared to treatment without the added approach. While this approach is beneficial, the method is also cost-effective, easy to perform, and has the potential to be useful in most clinical settings with minimal resources, making it an ideal option for inclusion into routine rehabilitation programs in order to facilitate improved upper limb function. More research involving larger subject groups and long-term follow-up is recommended to validate and extend these results.
